# Gene regulatory network inference by point-based Gaussian approximation filters incorporating the prior information

**DOI:** 10.1186/1687-4153-2013-16

**Published:** 2013-12-17

**Authors:** Bin Jia, Xiaodong Wang

**Affiliations:** 1 Intelligent Fusion Technology, Germantown, MD 20876, USA; 2 Department of Electrical Engineering, Columbia University, New York, NY 10027, USA

**Keywords:** Gene regulatory network, Point-based Gaussian approximation filters, Network prior information, Sparsity, Iterative thresholding

## Abstract

The extended Kalman filter (EKF) has been applied to inferring gene regulatory networks. However, it is well known that the EKF becomes less accurate when the system exhibits high nonlinearity. In addition, certain prior information about the gene regulatory network exists in practice, and no systematic approach has been developed to incorporate such prior information into the Kalman-type filter for inferring the structure of the gene regulatory network. In this paper, an inference framework based on point-based Gaussian approximation filters that can exploit the prior information is developed to solve the gene regulatory network inference problem. Different point-based Gaussian approximation filters, including the unscented Kalman filter (UKF), the third-degree cubature Kalman filter (CKF_3_), and the fifth-degree cubature Kalman filter (CKF_5_) are employed. Several types of network prior information, including the existing network structure information, sparsity assumption, and the range constraint of parameters, are considered, and the corresponding filters incorporating the prior information are developed. Experiments on a synthetic network of eight genes and the yeast protein synthesis network of five genes are carried out to demonstrate the performance of the proposed framework. The results show that the proposed methods provide more accurate inference results than existing methods, such as the EKF and the traditional UKF.

## 1 Introduction

Inferring gene regulatory network (GRN) has become one of the most important missions in system biology. Genome-wide expression data is widely used due to the development of several high-throughput experimental technologies. The gene regulatory network can be inferred from a number of gene expression samples taken over a period of time. Modeling of GRN is required before its structure can be inferred. Common dynamical modeling methods of GRN include Bayesian networks [1], Boolean networks [2], ordinary differential equations [3], state-space models [4,5], and so on. Various approaches based on different models have been used to infer the network from observed gene expression data, such as the Markov Chain Monte Carlo (MCMC) methods for the dynamic Bayesian network model [6] and the ordinary differential equation model [7], as well as the Kalman filtering methods for the state-space model [4,8] and the ordinary differential equation model [3]. Some survey papers can be found in [9-12].

Due to the ‘stochastic’ nature of the gene expression, the Kalman filtering approach based on the state-space model is one of the most competitive methods for inferring the GRN. The Kalman filter is optimal for linear Gaussian systems. However, the GRN is generally highly nonlinear. Hence, advanced filtering methods for nonlinear dynamic systems should be considered. The extended Kalman filter (EKF) is probably the most widely used nonlinear filter which uses the first-order Taylor series expansion to linearize the nonlinear model. However, the accuracy of the EKF is low when the system is highly nonlinear or contains large uncertainty. The point-based Gaussian approximation filters have been recently proposed to improve the performance of the EKF, which employ various quadrature rules to compute the integrals involved in the exact Bayesian estimation. Many filters fall into this category, such as the unscented Kalman filter (UKF) [13], the Gauss-Hermite quadrature filter [14], the cubature Kalman filter (CKF) [15], and the sparse-grid quadrature filter [16]. Besides the point-based Gaussian approximation filters, the particle filter has drawn much attention recently [17]. The particle filter uses random particles with weights to represent the probability density function (pdf) in the Bayesian estimation and provides better estimation result than the EKF. The main problem of the particle filter is that the computational complexity is high, and therefore, it is hard to use for high-dimensional problems, such as the problem considered in this paper.

The EKF and the particle filter have been used for the inference of GRN [4,8,18]. In this paper, we consider the point-based Gaussian approximation filters. Our main objective is to provide a framework of incorporating network prior information into the filters. For example, some gene regulations may be known [19] from literature and the inference accuracy of GRN can be improved by incorporating the known regulations of the GRN [20]. Integration of the prior knowledge or constraints with the GRN inference algorithm has been introduced to improve the inference result. The DNA motif sequence in gene promoter regions is incorporated in [21] while modeling of transcription factor interactions is incorporated in [22]. As mentioned in [20], experimentally determined physical interactions can be obtained. In addition, the sparsity constraint is frequently used in the inference of the GRN. To the best of the authors’ knowledge, the most related work in incorporating the prior information in Bayesian filters is [8]. In that work, rather than directly getting the inference results from the filter, an optimization method is used. In particular, a cost function is used in which the sparsity constraint is enforced. However, the cost function in [8] does not consider the uncertainty of the state in the filtering. That cost function in fact is not coupled well with the filtering algorithm. In addition, it did not consider other kinds of prior information. In this paper, we propose a new framework that incorporates the prior information effectively in the filtering algorithm by solving a constrained optimization problem. Efficient recursive algorithms are provided to solve the associated optimization problem.

The remainder of this paper is organized as follows. In Section 2, the modeling of gene regulatory network is introduced. The point-based Gaussian approximation filters are briefly introduced in Section 3. The proposed new filtering framework is described in Section 4. In Section 5, experimental results are provided. Finally, concluding remarks are given in Section 6.

## 2 State-space modeling of gene regulatory network

The GRN can be described by a graph in which genes are viewed as nodes and edges depict causal relations between genes. The structure of GRN reveals the mechanisms of biological cells. Analyzing the structure of GRN will pave the way for curing various diseases [23]. The learning of GRN has drawn much attention recently due to the availability the microarray data. By analyzing collected gene expression levels over a period of time, one can identify various regulatory relations between different genes. To facilitate the analysis of the GRN, modeling of GRN is required. Different models can be used, such as Bayesian networks [1], Boolean networks [2], ordinary differential equation [3], and state-space model [4,5]. The state-space model has been widely used because it incorporates noise and can make use of computationally efficient filtering algorithms [5]. Thus, we also use the state-space modeling of GRN in this paper.

Under the discrete-time state-space modeling of the gene regulatory networks, the network evolution from time *k* to time *k* - 1 can be described by

(1) $$x_{k}=f(x_{k-1})+v_{k},$$

where ***x***_*k *_= [ *x*_1,*k*_,…,*x*_*n*,*k*_]^*T*^ is the state vector and *x*_*i*,*k*_ denotes the gene expression level of the *i*-th gene at time *k*. ***f*** is a nonlinear function that characterizes the regulatory relationship among the genes. ***v***_*k*_ is the state noise and it is assumed to follow a Gaussian distribution with mean ***0*** and covariance matrix ***Q***_*k*_, i.e., $v_{k}∼N(0,Q_{k})$.

Following [8], we use the following nonlinear function in the state Equation (1):

(2) f(x)=Ag(x),

with

(3) $$g(x)=\left[\begin{array}{c} g_{1}(x_{1})\\ \vdots \\ g_{n}(x_{n}) \end{array}\right]$$

and

(4) $$g_{i}(x)=\frac{1}{1+e^{-\mu_{i}x}}.$$

In (2), ***A*** is the regulatory coefficient matrix with *a*_*ij*_ denoting the regulation coefficient from gene *j* to gene *i*. Note that a positive coefficient *a*_*ij*_ indicates that gene *j* activates gene *i* and a negative *a*_*ij*_ indicates that gene *j* represses gene *i*. In (4), *μ*_*i*_ is a parameter. Note that ***A*** and *μ*_*i*_ are unknown parameters. The discrete-time nonlinear stochastic dynamic system [24] shown in Eqs. (1)-(3) have been successfully used to describe the GRN [4,8]. Equation (4) is also called Sigmoid function which is frequently used since it is consistent with the fact that all concentrations get saturated at some point in time [25]. The Sigmoid function has been used in modeling GRN to verify various methods, such as artificial neural network [26], simulated annealing and clustering algorithm [27], extended Kalman filter [4], particle filter [8], and Genetic programming and Kalman filtering [25].

For the measurement model, we consider the following general nonlinear observation equation

(5) $$\begin{array}{l} y_{k}=h(x_{k})+n_{k}, \end{array}$$

where ***h*** (·) is some nonlinear function, ***n***_*k*_ is the measurement noise, which is assumed to follow the Gaussian distribution with mean ***0*** and covariance matrix ***R***_*k*_, i.e., $n_{k}∼N(0,R_{k})$. For example, if the noise corrupted expression levels are observed, then ***h***(***x***) = ***x***.

## 3 Network inference using point-based Gaussian approximation filters

### 3.1 Gaussian approximation filters

In this section, the framework of point-based Gaussian approximation filters for the state-space dynamic model is briefly reviewed. We consider the state-space model consisting of the state Equation (1) and the measurement Equation (5). We denote $y^{k}≜\;[\;y_{1},\ldots ,y_{k}]$.

The optimal Bayesian filter is composed of two steps: prediction and filtering. Specifically, given the prior pdf $p(x_{k-1}|y^{k-1})$ at time *k* - 1, the predicted conditional pdf $p(x_{k}|y^{k-1})$ is given by

(6) $$p(x_{k}|y^{k-1})=\int \;p(x_{k}|x_{k-1})p(x_{k-1}|y^{k-1})dx_{k-1}.$$

After the measurement at time *k* becomes available, the filtered pdf is given by

(7) $$p(x_{k}|y^{k})=\frac{p(y_{k}|x_{k})p(x_{k}|y^{k-1})}{\int p(y_{k}|x_{k})p(x_{k}|y^{k-1})dx_{k}}.$$

The pdf recursions in (6) and (7) are in general computationally intractable unless the system is linear and Gaussian. The Gaussian approximation filters approximate (6) and (7) by invoking Gaussian assumptions. Specifically, the first assumption is that given ***y***^*k*-1^, ***x***_*k*-1_ has a Gaussian distribution, i.e., $x_{k-1}|y^{k-1}∼N({\hat{x}}_{k-1|k-1},P_{k-1|k-1})$. The second assumption is that (***x***_*k*_,***y***_*k*_) are jointly Gaussian given ***y***^*k*-1^.

It then follows from the second assumption that given ***y***^*k*-1^, ***x***_*k*_ has a Gaussian distribution, i.e., $x_{k}|y^{k-1}∼N({\hat{x}}_{k|k-1},P_{k|k-1}).$ Using (1) and the first assumption, we have the predicted mean ${\hat{x}}_{k|k-1}$ and covariance ***P***_*k*|*k*-1_ given respectively by

(8) $$\begin{array}{lll} {\hat{x}}_{k|k-1} & ≜E\{x_{k}|y^{k-1}\}=E_{x_{k-1}|y^{k-1}}\left\{\;f(x_{k-1})\right\} & \\ & =\int f(x)\phi (x;\;{\hat{x}}_{k-1|k-1},P_{k-1|k-1})dx, & \end{array}$$

and

(9) $$\begin{array}{lll} P_{k|k-1} & ≜\;\text{Cov}\{x_{k}|y^{k-1}\}\; & \\ & =\;E_{x_{k-1}|y^{k-1}}\{\left(\;f(x_{k-1})-{\hat{x}}_{k|k-1}\right)\; & \\ & \;\times {\left(\;f(x_{k-1})-{\hat{x}}_{k|k-1}\right)}^{T}\}+Q_{k-1}\; & \\ & =\;\int (\;f(x)-{\hat{x}}_{k|k-1})\; & \\ & \;\times {\left(\;f(x)-{\hat{x}}_{k|k-1}\right)}^{T}\phi (x;\;{\hat{x}}_{k-1|k-1},\; & \\ & \;P_{k-1|k-1})dx+Q_{k-1},\; \end{array}$$

where $\phi \left(x;\;\hat{x},P\right)$ denotes the multivariate Gaussian pdf with mean $\hat{x}$ and covariance ***P***.

Then, following the second assumption, given $y^{k}=\;[y^{\;k-1},y_{k}]$, ***x***_*k*_ is Gaussian distributed, i.e., $x_{k}|y^{k}∼N({\hat{x}}_{k|k},P_{k|k}).$ Using the conditional property of the multivariate Gaussian distribution, the filtered mean ${\hat{x}}_{k|k}$ and covariance ***P***_*k*|*k*_ are given respectively by

(10) $$\begin{array}{lll} {\hat{x}}_{k|k} & ≜E\{x_{k}|y_{k},y^{k-1}\}\; & \\ & ={\hat{x}}_{k|k-1}+L_{k}(y_{k}-{\hat{y}}_{k|k-1})\; \end{array}$$

(11) $$\begin{array}{lll} \text{and}\;P_{k|k} & ≜\text{Cov}\{x_{k}|y_{k},y^{k-1}\}\; & \\ & =P_{k|k-1}-L_{k}P_{k}^{\text{xy}},\; \end{array}$$

with

(12) $$\begin{array}{lll} \;{\hat{y}}_{k|k-1} & =E_{x_{k}|y^{k-1}}\left\{h(x_{k})\right\}\; & \\ & =\int h(x)\phi \left(x;\;{\hat{x}}_{k|k-1},P_{k|k-1}\right)dx,\; \end{array}$$

(13) $$\begin{array}{ll} L_{k} & =P_{k}^{\text{xy}}{\left(R_{k}+P_{k}^{\text{yy}}\right)}^{-1},\; \end{array}$$

(14) $$\begin{array}{lll} P_{k}^{\text{xy}} & =E_{x_{k}|y^{k-1}}\left\{(x-{\hat{x}}_{k|k-1}){\left(h(x)-{\hat{y}}_{k|k-1}\right)}^{T}\right\}\; & \\ & =\int (x-{\hat{x}}_{k|k-1}){\left(h(x)-{\hat{y}}_{k|k-1}\right)}^{T}\; & \\ & \;\phi \left(x;\;{\hat{x}}_{k|k-1},P_{k|k-1}\right)dx,\; \end{array}$$

(15) $$\begin{array}{lll} P_{k}^{\text{yy}} & =E_{x_{k}|y^{k-1}}\left\{(h(x)-{\hat{y}}_{k|k-1}){\left(h(x)-{\hat{y}}_{k|k-1}\right)}^{T}\right\}\; & \\ & =\int \left(h(x)-{\hat{y}}_{k|k-1}\right){\left(h(x)-{\hat{y}}_{k|k-1}\right)}^{T}\; & \\ & \;\phi \left(x;{\hat{x}}_{k|k-1},P_{k|k-1}\right)dx.\; \end{array}$$

### 3.2 Point-based Gaussian approximation filters

The integrals in (8), (9), (12), (14) and (15) are Gaussian type that can be efficiently approximated by various quadrature methods. Specifically, if a set of weighted points $\left\{(\gamma_{i},w_{i}),i=1,\ldots ,N\right\}$ can be used to approximate the integral

(16) $$\int h(x)\;\phi \left(x;0,I\right)dx\approx \overset{N}{\underset{i=1}{\sum }}w_{i}h(\gamma_{i}),$$

then the general Gaussian-type integral can be approximated by

(17) $$\int h(x)\phi \left(x;\hat{x},P\right)dx\approx \overset{N}{\underset{i=1}{\sum }}w_{i}h(S\gamma_{i}+\hat{x}),$$

where ***P ***= ***SS***^*T*^ and ***S*** can be obtained by Cholesky decomposition or singular value decomposition (SVD).

Using (17), we can then approximate (8) and (9) as follows:

(18) $${\hat{x}}_{k|k-1}\approx \overset{N}{\underset{i=1}{\sum }}w_{i}\;f\left(\xi_{k-1,i}\right)$$

and

(19) $$\begin{array}{lll} P_{k|k-1}\approx & \overset{N}{\underset{i=1}{\sum }}w_{i}\;f\left(\xi_{k-1,\;i}-{\hat{x}}_{k|k-1}\right)\; & \\ & \times {\left(\xi_{k-1,\;i}-{\hat{x}}_{k|k-1}\right)}^{T}+Q_{k-1},\; \end{array}$$

where ***ξ***_*k* - 1,*i*_ is the transformed quadrature point obtained from the covariance decomposition, i.e.,

(20) $$\begin{array}{ll} P_{k-1|k-1} & =S_{k-1}S_{k-1}^{T},\; \end{array}$$

(21) $$\begin{array}{ll} \xi_{k-1,i} & =S_{k-1}\gamma_{i}+{\hat{x}}_{k-1|k-1}.\; \end{array}$$

Similarly, we can approximate (12), (14) and (15) as follows:

(22) $$\begin{array}{ll} \;{\hat{y}}_{k|k-1} & =\overset{N}{\underset{i=1}{\sum }}w_{i}h\left({\tilde{\xi }}_{k,i}\right),\; \end{array}$$

(23) $$\begin{array}{ll} P_{k}^{\text{xy}} & =\overset{N}{\underset{i=1}{\sum }}w_{i}\left({\tilde{\xi }}_{k,i}-{\hat{x}}_{k|k-1}\right){\left(h({\tilde{\xi }}_{k,i})-{\hat{y}}_{k|k-1}\right)}^{T},\; \end{array}$$

(24) $$\begin{array}{ll} P_{k}^{\text{yy}} & =\overset{N}{\underset{i=1}{\sum }}w_{i}\left(h({\tilde{\xi }}_{k,i})-{\hat{y}}_{k|k-1}\right){\left(h({\tilde{\xi }}_{k,i})-{\hat{y}}_{k|k-1}\right)}^{T},\; \end{array}$$

where ${\tilde{\xi }}_{k,i}$ is the transformed point obtained from the decomposition of the predicted covariance, i.e.,

(25) $$\begin{array}{ll} P_{k|k-1} & ={\tilde{S}}_{k}{\tilde{S}}_{k}^{T},\; \end{array}$$

(26) $$\begin{array}{ll} {\tilde{\xi }}_{k,i} & ={\tilde{S}}_{k}\gamma_{i}+{\hat{x}}_{k|k-1}.\; \end{array}$$

Various numerical rules can be used to form the approximation in (16), which lead to different Gaussian approximation filters. In particular, the unscented transformation, the Gauss-Hermite quadrature rule, and the sparse-grid quadrature rules are used in the unscented Kalman filter (UKF), the Gauss-Hermite quadrature Kalman filter (GHQF), and the sparse-grid quadrature filter (SGQF), respectively.

Recently, the fifth-degree quadrature filter has been proposed and shown to be more accurate than the third-degree quadrature filters, such as the UKF and the third-degree cubature Kalman filter (CKF_3_), when the system is highly nonlinear or contains large uncertainty [16]. In this paper, we consider the UKF, CKF_3_, and the fifth-degree cubature Kalman filter (CKF_5_). Other filters such as the central difference filter [14] and divided difference filter [28] can also be used. The CKF_5_ is based on Mysovskikh’s method which uses fewer point than the fifth-degree quadrature filter in [16]. In the following, different numerical rules used in (16) are briefly summarized.

#### ***3.2.1 Unscented transform***

In the unscented Kalman filter (UKF), we have *N* = 2 *n* + 1 where *n* is the dimension of ***x***. The quadrature points and the corresponding weights are given respectively by

(27) $$\;\gamma_{i}=\{\begin{array}{ll} 0,\; & i=1,\\ \sqrt{\left(n+\kappa \right)}e_{i-1},\; & i=2,\cdots \;,n+1,\\ -\sqrt{\left(n+\kappa \right)}e_{i-n-1},\; & i=n+2,\cdots \;,2n+1, \end{array}$$

and

(28) $$w_{i}=\{\begin{array}{ll} \frac{\kappa }{n+\kappa },\; & i=1,\\ \frac{1}{2(n+\kappa )},\; & i=2,\cdots \;,2n+1, \end{array}$$

where *κ* is a tunable parameter, and ***e***_*i*_ is the *i*-th *n*-dimensional unit vector in which the *i*-th element is 1 and other elements are 0.

#### ***3.2.2 Cubature rules***

The left-hand side of (16) can be rewritten as

(29) $$\;\int h(x)\;\phi \left(x;0,I\right)dx=\frac{1}{\pi^{n/2}}\int h\left(\sqrt{2}x\right)\text{exp}\left(-x^{T}x\right)dx.$$

Consider the integral

(30) $$I\left(h\right)=\int h(x)\text{exp}\left(-x^{T}x\right)dx.$$

By letting ***x*** = *r****s*** with ***s***^*T*^***s*** = 1 and $r=\sqrt{x^{T}x}$, *I*(***h***) can be rewritten in the spherical-radial coordinate system as

(31) $$I\left(h\right)=\overset{\infty }{\underset{0}{\int }}\;\underset{U_{n}}{\int }h(rs)r^{n-1}\text{exp}\left(-r^{2}\right)d\sigma \left(s\right)dr,$$

where $U_{n}=\left\{s\in R^{n}:∥s∥=1\right\}$, and $\sigma \left(\cdot \right)$ is the spherical surface measure or the area element on *U*_*n*_.

Note that (31) contains two types of integrals: the radial integral $\overset{\infty }{\underset{0}{\int }}h_{r}\left(r\right)r^{n-1}\text{exp}\left(-r^{2}\right)dr$ and the spherical integral $\underset{U_{n}}{\int }h_{s}(s)d\sigma (s)$.

If the radial rule can be approximated by

(32) $$\overset{\infty }{\underset{0}{\int }}\;h_{r}(r)r^{n-1}\text{exp}\left(-r^{2}\right)dr\approx \overset{N_{r}}{\underset{i=1}{\sum }}w_{r,i}h_{r}(r_{i}),$$

and the spherical integral can be approximated by

(33) $$\underset{U_{n}}{\int }h_{s}(s)d\sigma (s)\approx \overset{N_{s}}{\underset{j=1}{\sum }}w_{s,j}h_{s}(s_{j}),$$

then (31) can be approximated by

(34) $$\begin{array}{llll} I(h) & \approx & \overset{N_{r}}{\underset{i=1}{\sum }}\overset{N_{s}}{\underset{j=1}{\sum }}w_{r,i}w_{s,j}h(r_{i}s_{j}). & \end{array}$$

A third-degree cubature rule to approximate (29) is obtained by using the third-degree spherical rule and radial rule [15]:

(35) $$\;\int h(x)\phi \left(x;0,I\right)dx\approx \frac{1}{2n}\overset{n}{\underset{i=1}{\sum }}\left[h\left(\sqrt{n}e_{i}\right)+h\left(-\sqrt{n}e_{i}\right)\right].$$

*Remark:* The third-degree cubature rule is identical to the unscented transformation with *κ* = 0.

To construct the fifth-degree cubature rule, the Mysovskikh’s method [29] and the moment matching method [16] are used to provide the fifth-degree spherical rule and radial rule, respectively. The final fifth-degree cubature rule is given by

(36) $$\begin{array}{ll} \;\int \;\;h(x)\phi \left(x;0,I\right)dx\approx & \frac{2}{n+2}h(0)+\\ & +\frac{n^{2}(7-n)}{2{\left(n+1\right)}^{2}{\left(n+2\right)}^{2}}\overset{n+1}{\underset{i=1}{\sum }}[h\left(\sqrt{n+2}s_{1}^{\left(i\right)}\right)\\ & +\;h\left(-\sqrt{n+2}s_{1}^{\left(i\right)}\right)]\\ & +\frac{2{\left(n-1\right)}^{2}}{{\left(n+1\right)}^{2}{\left(n+2\right)}^{2}}\;\overset{n(n+1)/2}{\underset{i=1}{\sum }}\;[h\;\left(\sqrt{n\;+\;2}s_{2}^{\left(i\right)}\right)\\ & +\;h\left(-\sqrt{n+2}s_{2}^{\left(i\right)}\right)]\;, \end{array}$$

where the point $s_{1}^{\left(i\right)}$ is given by

(37) $$s_{1}^{\left(i\right)}=\left[p_{1}^{\left(i\right)},p_{2}^{\left(i\right)},\cdots \;,p_{n}^{\left(i\right)}\right],\;i=1,2,\cdots \;,n+1,$$

with

(38) $$p_{j}^{\left(i\right)}=\{\begin{array}{ll} -\sqrt{\frac{n+1}{n(n-j+2)(n-j+1)}},\; & j<i,\\ \sqrt{\frac{\left(n+1)(n-i+1\right)}{n(n-i+2)}},\; & j=i,\\ 0,\; & j>\mathrm{i.} \end{array}$$

Moreover, the set of points $\left\{s_{2}^{\left(i\right)}\right\}$ is given by

(39) $$\begin{array}{lll} \left\{s_{2}^{\left(i\right)}\right\}= & \{\sqrt{\frac{n}{2(n-1)}}\left(s_{1}^{\left(k\right)}+s_{1}^{\left(l\right)}\right):k<l,\;k,l=1,\; & \\ & \;2,\cdots \;,n+1\}.\; \end{array}$$

### 3.3 Augmented state-space model for network inference

In the state-space model for gene regulatory networks described in Section 3.2, the underlying network structure is characterized by the *n* × *n* regulatory coefficient matrix ***A*** in (2) and the parameters ***μ*** = [ *μ*_1_,…,*μ*_*n*_] in (4). The problem of network inference then becomes to estimate ***A*** and ***μ***. To do that, we incorporate the unknown parameters ***A*** and ***μ*** into the state vector to obtain an augmented state-space model, and then apply the point-based Gaussian approximation filters to estimate the space vector and thereby obtaining the estimates of ***A*** and ***μ***.

Specifically, we denote $\theta =\left[a_{11},a_{12},\cdots \;,a_{1n},\cdots \;,\right){\left(a_{\text{nn}},\mu_{1},\cdots \;,\mu_{n}\right]}^{T}$ and the augmented state vector ${\bar{x}}_{k}={\left[x_{k}^{T},\theta^{T}\right]}^{T}$. Then, the augmented state equation can be written as

(40) $${\bar{x}}_{k}=\bar{f}({\bar{x}}_{k-1})+{\bar{v}}_{k}=\left[\begin{array}{l} A_{k-1}\;g_{k-1}(x_{k-1})\\ \theta_{k-1} \end{array}\right]+\left[\begin{array}{l} v_{k-1}\\ 0 \end{array}\right].$$

Note that ***A***_*k*-1_ and ***g***_*k*-1_ can be obtained from ***θ***_*k*-1_, and ${\bar{v}}_{k}∼N(0,{\bar{Q}}_{k})$ with ${\bar{Q}}_{k}=\text{diag}\;\left(\left[\;Q_{k}\;O_{n^{2}+n}\right]\right)$, where ***O***_*m*_ denotes an *m* × *m* all-zero matrix.

In the remainder of this paper, we assume that the noisy gene expression levels are observed. Therefore, the augmented measurement equation becomes

(41) $$y_{k}=h({\bar{x}}_{k})+n_{k}=B{\bar{x}}_{k}+n_{k},$$

where $B=\;[\;I_{n},O_{n\times (n^{2}+n)}]$, $O_{n\times (n^{2}+n)}$ denotes an *n* × (*n*^2^ + *n*) all zeros matrix.

The point-based Gaussian approximation filters can then be used to obtain the estimate of the augmented state, ${\hat{\bar{x}}}_{k}$, from which the estimates of the unknown network parameters, i.e., $\hat{A}$ and $\hat{\mu }$ can then be obtained.

Note that since the measurement Equation (41) is linear, the filtering Equations (10, 11) become

(42) $$\begin{array}{ll} {\hat{\bar{x}}}_{k|k} & ={\hat{\bar{x}}}_{k|k-1}+L_{k}(y_{k}-B{\hat{\bar{x}}}_{k|k-1}),\; \end{array}$$

(43) $$\begin{array}{ll} \text{and}\;P_{k|k} & =P_{k|k-1}-L_{k}BP_{k|k-1},\; \end{array}$$

(44) $$\begin{array}{ll} \text{with}\;L_{k} & =P_{k|k-1}B^{T}{\left(R_{k}+BP_{k|k-1}B^{T}\right)}^{-1},\; \end{array}$$

which are the same as the filtering updates for Kalman filters.

## 4 Incorporating prior information

In practice, some prior knowledge on the underlying GRN is typically available. In this section, we outline approaches to incorporating such prior knowledge into the point-based Gaussian approximation filters for network inference. In particular, we consider two types of prior information, namely, sparsity constraints and range constraints on the network. For networks with sparsity constraints, we incorporate an iterative thresholding procedure into the Gaussian approximation filters. And to accommodate range constraints, we employ PDF-truncated Gaussian approximation filters.

### 4.1 Optimization-based approach for sparsity constraints

#### ***4.1.1 The optimization formulations***

Note that under the Gaussian assumption, the state estimation ${\hat{\bar{x}}}_{k|k}$ of the Kalman filter is equivalently given by the solution to the following optimization problem [30,31]

(45) $$\begin{array}{ll} {\hat{\bar{x}}}_{k|k} & =\underset{\bar{x}}{\text{arg}\;\text{min}}\;J(\bar{x}),\; \end{array}$$

(46) $$\begin{array}{lll} \text{with}\;J\;(\bar{x}) & ≜{\left(y_{k}-h(\bar{x})\right)}^{T}R_{k}^{-1}\left(y_{k}-h(\bar{x})\right)\; & \\ & \;+{\left(\bar{x}-{\hat{\bar{x}}}_{k|k-1}\right)}^{T}P_{k|k-1}^{-1}\left(\bar{x}-{\hat{\bar{x}}}_{k|k-1}\right).\; \end{array}$$

To incorporate the prior information of the GRN, (46) is modified as

(47) $$\tilde{J}(\bar{x})=J(\bar{x})+\lambda \;J_{p}(\bar{x}),$$

where $J_{p}(\bar{x})$ is a penalty function associated with the prior information and *λ* is a tunable parameter that regulates the tightness of the penalty.

For example, in gene regulatory networks, each gene only interacts with a few genes [20]. To capture such a sparsity constraint, a Laplace prior distribution can be used for the connection coefficient matrix ***A***, i.e.,

(48) $$p(A)={\left(\lambda /2\right)}^{n^{2}}\text{exp}\left(-\lambda \overset{n}{\underset{i=1}{\sum }}\overset{n}{\underset{j=1}{\sum }}|a_{\text{ij}}|\right).$$

Therefore, in this case, $J_{p}(\bar{x})\;=\;-\text{log}p(A)\;=\;c_{1}∥A{∥}_{1}+c_{2}$ where *c*_1_ and *c*_2_ are constants. And, (47) can be rewritten as

(49) $$\tilde{J}(\bar{x})=J(\bar{x})+\lambda ∥A{∥}_{1}.$$

Note that (49) can also be interpreted as the result of applying the least squares shrinkage selection operator (LASSO) to (47). The LASSO adds an *L*_1_ - norm constraint to the GRN so that the regulatory coefficient matrix ***A*** tends to be sparse with many zero elements.

As another example, if some known regulatory relationship exists, then it should be taken into account to improve the estimation accuracy. Specifically, define an *n* × *n* indicator matrix ***E*** = [ *e*_*i*,*j*_] where *e*_*ij*_ = 1 indicates that there is a lack of regulation from gene *j* to gene *i*. Then, similar to the use of LASSO, a penalty on *a*_*ij*_ should incur if *e*_*ij*_ = 1. Thus, (47) can be rewritten as

(50) $$\tilde{J}(\bar{x})=J(\bar{x})+\lambda ∥E\circ A{∥}_{1}.$$

Note that as in [20], here we do not force *a*_*ij*_ = 0 corresponding to *e*_*ij*_ = 1 but rather use an *L*_1_ - norm penalty. The advantage of such an approach is that it allows the algorithm to pick different structures but more likely to pick the edges without penalties. ‘o’ denotes the entry-wise product operation of two matrices.

#### ***4.1.2 Iterative thresholding algorithm***

Solving the optimization problems in (49) and (50) is not straightforward since |*a*| is non-differentiable at *a* = 0. In the following, an efficient solver called the iterative thresholding algorithm is introduced.

For convenience, we consider a general optimization problem of the form

(51) $$\underset{\bar{x}}{\text{arg}\;\text{min}}\;J(\bar{x})=L(\bar{x})+∥\text{λ}\circ \bar{x}{∥}_{1},$$

where $\text{λ}={\left[\;\lambda_{1},\lambda_{2},\cdots \;,\lambda_{n^{2}+2n}\right]}^{T}$ and $L(\bar{x})$ is a smooth function. Note that if $\text{λ}=\;{\left[\;0_{1\times n},\lambda \times 1_{1\times n^{2}},0_{1\times n}\right]}^{T}$, then (51) becomes (49); and if $\text{λ}=\;{\left[\;0_{1\times n},\lambda \times \hat{\underline{\theta }},0_{1\times n}\right]}^{T}$, then (51) becomes (50). Note that $\hat{\underline{\theta }}=\;{\left[e_{11},e_{12},\cdots \;,e_{1n},\cdots \;,e_{\text{nn}}\right]}^{T}$.

The solution to (51) can be iteratively obtained by solving a sequence of optimization problems. As in Newton’s method, the Taylor series expansion of $L(\bar{x})$ around the solution ${\bar{x}}^{t}$ at the *t*-th iteration is given by

(52) $$L({\bar{x}}^{t}+\Delta \bar{x})≅L({\bar{x}}^{t})+\Delta {\bar{x}}^{T}\nabla L({\bar{x}}^{t})+\frac{\alpha_{t}}{2}∥\Delta \bar{x}{∥}_{2}^{2},$$

where ∇*L* is the gradient of *L* and *α*_*t*_ is such that *α*_*t*_***I*** mimics the Hessian ∇^2^*L*. Then, ${\bar{x}}^{t+1}$ is given by [32]

(53) $${\bar{x}}^{t+1}=\underset{z}{\text{arg}\;\text{min}}\;{\left(z-{\bar{x}}^{t}\right)}^{T}\nabla L({\bar{x}}^{t})+\frac{\alpha_{t}}{2}∥z-{\bar{x}}^{t}{∥}_{2}^{2}+∥\text{λ}\circ z{∥}_{1}.$$

The equivalent form of (53) is given by [32]

(54) $$\begin{array}{ll} {\bar{x}}^{t+1} & =\underset{z}{\text{arg}\;\text{min}}\;\frac{1}{2}∥z-u^{t}{∥}_{2}^{2}+\frac{1}{\alpha_{t}}∥\text{λ}\circ z{∥}_{1},\; \end{array}$$

(55) $$\begin{array}{ll} \text{with}\;u^{t} & ={\bar{x}}^{t}-\frac{1}{\alpha_{t}}\nabla L({\bar{x}}^{t}),\; \end{array}$$

(56) $$\begin{array}{llll} \alpha_{t} & \approx \; & \frac{{\left(s^{t}\right)}^{T}r^{t}}{∥s^{t}{∥}^{2}}, & \end{array}$$

(57) $$\begin{array}{ll} s^{t} & ={\bar{x}}^{t}-{\bar{x}}^{t-1},\; \end{array}$$

(58) $$\begin{array}{ll} r^{t} & =\nabla L({\bar{x}}^{t})-\nabla L({\bar{x}}^{t-1}).\; \end{array}$$

The solution to (54) is given by [32]${\bar{x}}^{t+1}=\eta^{S}(u^{t},\frac{\text{λ}}{\alpha_{t}})$, where

(59) $$\eta^{S}(u,a)=\text{sign}(u)\text{max}\left\{|u|-a,0\right\}$$

is the soft thresholding function with sign(***u***) and $\text{max}\left\{|u|-a,0\right\}$ being component-wise operators.

Finally, the iterative procedure for solving (51) is given by

(60) $$\;{\bar{x}}^{t+1}=\text{sign}\left(\;{\bar{x}}^{t}-\frac{1}{\alpha_{t}}\nabla L({\bar{x}}^{t})\;\right)\text{max}\;\left\{\left|{\bar{x}}^{t}-\frac{1}{\alpha_{t}}\nabla L({\bar{x}}^{t})\right|-\frac{\text{λ}}{\alpha_{t}},0\right\}\;.$$

And the iteration stops when the following condition is met

(61) $$\frac{\left|J({\bar{x}}^{t})-J({\bar{x}}^{t-1})\right|}{\left|J({\bar{x}}^{t-1})\right|}\leq \varepsilon ,$$

where *ε* is a given small number.

### 4.2 PDF truncation method for range constraints

If the range constraints on the regulatory coefficients are available, the inference accuracy can be improved by enforcing the constraints in the Gaussian approximation filters.

In particular, assume that we impose the following range constraints on the state vector $\bar{x}$

(62) $$c\leq \bar{x}\leq d.$$

The PDF truncation method [31] can be employed to incorporate the above range constraint into the Gaussian approximation filters, by converting the updated mean ${\hat{\bar{x}}}_{k|k}$ and covariance ***P***_*k*|*k*_ to the pseudo mean ${\hat{\bar{x}}}_{k|k}^{t}$ and covariance $P_{k|k}^{t}$ which are then used in the next prediction and filtering steps.

We next briefly outline the PDF truncation procedure. We use ${\hat{\bar{x}}}_{k|k,i}^{t}$ and $P_{k|k,i}^{t}$ to denote the mean and covariance after the first *i* constraints have been enforced. Initially, we set ${\hat{\bar{x}}}_{k|k,0}^{t}={\hat{\bar{x}}}_{k|k}$ and $P_{k|k,0}^{t}=P_{k|k}$. Consider the following transformation

(63) $$z_{k,i}=G_{i}D_{i}^{-1/2}S_{i}^{T}({\bar{x}}_{k}-{\hat{\bar{x}}}_{k|k,i}^{t})$$

where $S_{i}$ and $D_{i}$ are obtained from the Jordan canonical decomposition $S_{i}D_{i}S_{i}^{T}=P_{k|k,i}^{t}$ and $G_{i}$ is obtained by using the Gram-Schmidt orthogonalization and it satisfies [33]

(64) $$G_{i}D_{i}^{1/2}S_{i}^{T}e_{i}=\left[{\left(e_{i}^{T}P_{k|k,i}^{t}e_{i}\right)}^{1/2},0,\cdots \;,0\right]\;.$$

Then, the upper bound $e_{i}^{T}\bar{x}\leq d_{i}$ is transformed to [33]

(65) $$[\;1,0,\cdots \;,0]z_{k,i}\leq \frac{d_{i}-e_{i}^{T}{\hat{\bar{x}}}_{k|k,i}^{t}}{{\left(e_{i}^{T}P_{k|k,i}^{t}e_{i}\right)}^{1/2}}≜{\tilde{d}}_{i}.$$

Similarly, the lower bound $e_{i}^{T}\bar{x}\geq c_{i}$ is transformed to

(66) $$[\;1,0,\cdots \;,0]z_{k,i}\geq \frac{c_{i}-e_{i}^{T}{\hat{\bar{x}}}_{k|k,i}^{t}}{{\left(e_{i}^{T}P_{k|k,i}^{t}e_{i}\right)}^{1/2}}≜{\tilde{c}}_{i}.$$

The constraint requires that the first element of ***z***_*k*,*i*_ lies between ${\tilde{c}}_{i}$ and ${\tilde{d}}_{i}$. Hence, only the truncated PDF of the first element of ***z***_*k*,*i*_ is considered and it is given by [33]

(67) $$\begin{array}{ll} f(z) & =\alpha_{i}\text{exp}(-z^{2}/2),\; \end{array}$$

(68) $$\begin{array}{ll} \text{with}\;\alpha_{i}\; & =\frac{\sqrt{2}}{\sqrt{\pi }[\;\text{erf}({\tilde{d}}_{i}/\sqrt{2})-\text{erf}({\tilde{c}}_{i}/\sqrt{2})]}.\; \end{array}$$

Then, the mean and variance of the first element of ***z***_*k*,*i*_ after imposing the *i*-th constraint are given respectively by

(69) $$\begin{array}{ll} \mu_{i}= & \overset{{\tilde{d}}_{i}}{\underset{{\tilde{c}}_{i}}{\int }}\text{zf}(z)dz=\alpha_{i}\left[\text{exp}(-{\tilde{c}}_{i}^{2}/2)-\text{exp}(-{\tilde{d}}_{i}^{2}/2)\right],\; \end{array}$$

(70) $$\begin{array}{lll} \sigma_{i}^{2}= & \overset{{\tilde{d}}_{i}}{\underset{{\tilde{c}}_{i}}{\int }}{\left(z-\mu_{i}\right)}^{2}f(z)dz\; & \\ = & \;\alpha_{i}[\text{exp}(-{\tilde{c}}_{i}^{2}/2)({\tilde{c}}_{i}-2\mu_{i})\; & \\ & -\;\text{exp}(-{\tilde{d}}_{i}^{2}/2)({\tilde{d}}_{i}-2\mu_{i})]+\mu_{i}^{2}+1.\; \end{array}$$

Thus, the mean and covariance of the transformed state vector after imposing the *i*-th constraint are given respectively by

(71) $$\begin{array}{ll} {\bar{z}}_{k,i} & ={\left[\mu_{i},0,\cdots \;,0\right]}^{T},\; \end{array}$$

(72) $$\begin{array}{ll} Q_{k,i} & =\;\text{diag}\;([\sigma_{i}^{2},1,\cdots \;,1]).\; \end{array}$$

By taking the inverse transform of (63), we then get

(73) $$\begin{array}{ll} {\hat{\bar{x}}}_{k|k,i+1}^{t} & =S_{i}D_{i}^{1/2}G_{i}^{T}{\bar{z}}_{k,i}+{\hat{\bar{x}}}_{k|k,i}^{t},\; \end{array}$$

(74) $$\begin{array}{ll} P_{k|k,i+1}^{t} & =S_{i}D_{i}^{1/2}G_{i}^{T}Q_{k,i}G_{i}D_{i}^{1/2}S_{i}^{T}.\; \end{array}$$

After imposing all *n* constraints, the final constrained state estimate and covariance at time *k* are given respectively by ${\hat{\bar{x}}}_{k|k}^{t}≜{\hat{\bar{x}}}_{k|k,n}^{t}$ and $P_{k|k}^{t}≜P_{k|k,n}^{t}$.

## 5 Numerical results

### 5.1 Synthetic network

In this section, a synthetic network that contains eight genes is used to test the performance of the EKF, the UKF, the CKF_3_, the CKF_5_, and their corresponding filters incorporating the prior information. Forty data points are collected to infer the structure of the network. The system noise and measurement noise are assumed to be Gaussian distributed with means ***0*** and covariances ${\bar{Q}}_{k}=\text{diag}\left(\right)[0.01I_{8}\;O_{72}]\;))$ and ***R***_*k*_ = 0.01 ***I***_8_, respectively. The connection coefficient matrix is given by

(75) $$\begin{array}{l} \;A=\left(\begin{array}{llllllll} 0 & 0 & 0 & 0 & 0 & 0 & 2.4 & 3.2\\ 0 & 0 & 0 & 4.1 & 0 & -2.4 & 0 & 4.1\\ -5.0 & 2.1 & -1.5 & 0 & 4.5 & 0 & 2.1 & 0\\ 0 & 1.3 & 2.5 & -3.7 & 1.8 & 0 & 0 & -3.1\\ 0 & 0 & 0 & -2.6 & -3.2 & 0 & -1 & 4\\ -1.5 & -1.8 & 0 & 3.4 & 1.4 & 1.1 & 0 & 1.7\\ -1.8 & 0 & 0 & -3 & 1.1 & 2.4 & 0 & 0\\ -1.3 & 0 & -1 & 0 & 2.1 & 0 & 0 & 2.2 \end{array}\right) \end{array}$$

and *μ*_*i*_ = 2, *i* = 1,⋯,8. For the filter, each coefficient in $\hat{A}$ is initialized from a Gaussian distribution with mean 0 and variance 0.2. Moreover, the coefficient *μ*_*i*_ is initialized from a Gaussian distribution with mean 1.5 and variance 0.2. The system state is initialized using the first measurement.

The metric used to evaluate the inferred GRN is the true positive rate (TPR), the false positive rate (FPR), and the positive predictive value (PPV). They are given by [34]

(76) $$\text{TPR}=\frac{\text{TP}\#}{\text{TP}\#+\text{FN}\#},$$

(77) $$\text{FPR}=\frac{\text{FP}\#}{\text{FP}\#+\text{TN}\#},$$

(78) $$\text{PPV}=\frac{\text{TP}\#}{\text{TP}\#+\text{FP}\#},$$

where the number of true positives (TP *#*) denotes the number of links correctly predicted by the inference algorithm; the number of false positives (FP *#*) denotes the number of incorrectly predicted links; the number of true negatives (TN *#*) denotes the number of correctly predicted nonlinks; and the number of false negatives (FN *#*) denotes the number of missed links by the inference algorithm [8].

#### ***5.1.1 Comparison of the EKF with point-based Gaussian approximation filters***

The UKF with different parameter *κ* is tested. The simulation results based on 50 Monte Carlo runs are shown in Table 1. It can be seen that UKFs with *κ*=0,2,5 have slight better performance than UKFs with *κ* = -5,-2. One possible reason is that the weights of all sigma points used in the UKF are all positive when *κ* ≥ 0. In general, all positive weights will guarantee better stability of the filtering algorithm. However, it should be emphasized that, in this specific example, there is no big difference between UKFs with different *κ*. In addition, the objective of this paper was to investigate the proposed filter incorporating the prior information. Hence, the UKF is used to denote UKF with *κ* = 3 - *n* and compare with the filters incorporating the prior information.

**Table 1:** **Comparison of UKF with different *κ***

	**True positive rate**						**False positive rate**			**Positive predictive rate**		
**Filters**	**Min**		**Max**		**Avg**		**Min**	**Max**	**Avg**	**Min**	**Max**	**Avg**
UKF(*κ* = -5)	0.7576		0.9355		0.8472		0.5000	0.7647	0.5955	0.5094	0.6279	0.5824
UKF(*κ* = -2)	0.7576		0.9355		0.8406		0.5161	0.7647	0.5933	0.5094	0.6279	0.5825
UKF(*κ* = 0)	0.7576		0.9375		0.8426		0.5161	0.7647	0.5918	0.5094	0.6364	0.5840
UKF(*κ* = 2)	0.7576		0.9375		0.8407		0.5152	0.7353	0.5895	0.5098	0.6279	0.5841
UKF(*κ* = 5)	0.7576		0.9063		0.8394		0.5161	0.7353	0.5933	0.5192	0.6279	0.5821

The inference results of the EKF, the UKF, the CKF_3_, and the CKF_5_ are summarized in Table 2, all results are based on 50 Monte Carlo runs. It can be seen that all point-based Gaussian approximation filters have better performance than the EKF since the average(avg) FPR is lower and the average TPR and precision are higher than that of the EKF. Although the CKFs exhibit slightly better filtering performance than the UKF in some runs, they are comparable in terms of TPR, FPR, and PPV.

**Table 2:** Comparison of different filters

		**True positives #**			**False positives #**			**True negatives #**			**False negatives** #	
**Filters**	**Min**	**Max**	**Avg**	**Min**	**Max**	**Avg**	**Min**	**Max**	**Avg**	**Min**	**Max**	**Avg**
EKF	2	17	10.60	23	44	36.4	2	15	7.08	2	24	9.92
UKF	25	29	26.80	16	26	19.28	8	16	13.06	2	8	4.86
CKF_3_	25	30	26.74	16	26	19.10	8	15	13.14	2	8	5.02
CKF_5_	25	29	26.64	16	26	19.24	8	16	13.08	1	8	5.04
	**True positive rate**						**False positive rate**			**Positive predictive rate**		
**Filters**	**Min**		**Max**		**Avg**		**Min**	**Max**	**Avg**	**Min**	**Max**	**Avg**
EKF	0.0769		0.8667		0.5224		0.6053	0.9545	0.8358	0.0800	0.3208	0.2231
UKF	0.7576		0.9355		0.8472		0.5	0.7576	0.5955	0.5094	0.6279	0.5824
CKF_3_	0.7576		0.9375		0.8426		0.5161	0.7647	0.5918	0.5094	0.6364	0.5840
CKF_5_	0.7576		0.9667		0.8417		0.5000	0.7647	0.5946	0.5094	0.6279	0.5814

Based on the above tests, in the rest of the paper, only the UKF is used.

#### ***5.1.2 Comparison of the UKF and the UKF incorporating the prior information***

As mentioned above, the UKF is used as a typical filter to compare the performance with and without the prior information.

##### 

**Incorporating existing network information** The following prior existing network information is assumed to be known: 1) gene1, gene5, and gene7 have little possibility to regulate gene2; 2) gene2, gene3, gene8 have little possibility to regulate gene7. Hence, the indicator matrix in (50) is given by

(79) $$\begin{array}{l} E=\left(\begin{array}{llllllll} 0 & 0 & 0 & 0 & 0 & 0 & 0 & 0\\ 1 & 0 & 0 & 0 & 1 & 0 & 1 & 0\\ 0 & 0 & 0 & 0 & 0 & 0 & 0 & 0\\ 0 & 0 & 0 & 0 & 0 & 0 & 0 & 0\\ 0 & 0 & 0 & 0 & 0 & 0 & 0 & 0\\ 0 & 0 & 0 & 0 & 0 & 0 & 0 & 0\\ 0 & 1 & 1 & 0 & 0 & 0 & 0 & 1\\ 0 & 0 & 0 & 0 & 0 & 0 & 0 & 0 \end{array}\right). \end{array}$$

The comparison of the UKF and the UKF incorporating the existing network information (denoted by UKF_*p*1_) with *λ* = 2 is shown in Table 3. It can be seen that the average TP *#* and TN *#* of the UKF_*p*1_ are both higher than those of the UKF. In addition, the average FP *#* and FN *#* of the UKF_*p*1_ are lower than those of the UKF. Hence, the UKF_*p*1_ predicts more correct links and nonlinks than the UKF. Moreover, the UKF_*p*1_ produces less incorrect links and missed links than the UKF. The average TPR and the precision of the UKF_*p*1_ are higher than those of the UKF. In addition, the average FPR of the UKF_*p*1_ is lower than that of the UKF. Hence, by using the existing network information, the inference accuracy can be improved.

**Table 3:** Inferred results of the conventional filter and filters incorporating the prior information

		**True positives #**			**False positives #**			**True negatives #**			**False negatives #**	
**Filters**	**Min**	**Max**	**Avg**	**Min**	**Max**	**Avg**	**Min**	**Max**	**Avg**	**Min**	**Max**	**Avg**
UKF	25	29	26.80	16	26	19.28	8	16	13.06	2	8	4.86
UKF_*p*1_	25	29	27.34	14	19	16.52	13	18	15.72	2	8	4.42
UKF_*p*2_	23	26	24.16	13	16	13.86	16	18	17.20	7	10	8.78
UKF_*p*3_	25	29	26.70	12	24	17.50	9	19	14.50	3	8	5.30
	**True positive rate**						**False positive rate**			**Positive predictive rate**		
**Filters**	**Min**		**Max**		**Avg**		**Min**	**Max**	**Avg**	**Min**	**Max**	**Avg**
UKF	0.7576		0.9355		0.8472		0.5	0.7647	0.5955	0.5094	0.6279	0.5824
UKF_*p*1_	0.7576		0.9355		0.8614		0.4375	0.5935	0.5121	0.5778	0.6744	0.6239
UKF_*p*2_	0.6970		0.7879		0.7335		0.4194	0.5000	0.4462	0.5897	0.6667	0.6355
UKF_*p*3_	0.7576		0.9063		0.8348		0.3871	0.7273	0.5463	0.5294	0.6923	0.6049

The performance of UKF_*p*1_ with different *λ* is shown in Table 4. It is seen that the performance of UKF_*p*1_ and UKF is close when *λ* is small since only small regulation is imposed on the solution. When *λ* is large, the difference between the UKF_*p*1_ and UKF is large. In particular, the UKF_*p*1_ provides sparser solution than the UKF when *λ* is large. It can be seen from Table 4, the average FPR of UKF_*p*1_ decreases with the increasing of *λ*. The average TPR of UKF_*p*1_, however, does not increase monotonically with the increasing of *λ*. The average PPR of UKF_*p*1_ increases with the increasing of *λ*. Hence, roughly speaking, the UKF_*p*1_ is better than the UKF when large *λ* is used.

**Table 4:** **Comparison of UKF **_***p1 ***_** using different *λ***

	**True positive rate**						**False positive rate**			**Positive predictive rate**		
**Filters**	**Min**		**Max**		**Avg**		**Min**	**Max**	**Avg**	**Min**	**Max**	**Avg**
UKF_*p*1 _(*λ * = 0.1)	0.7576		0.9355		0.8484		0.5000	0.7647	0.5900	0.5094	0.6279	0.5850
UKF_*p*1 _(*λ* = 0.5)	0.7576		0.9677		0.8535		0.4688	0.7647	0.5696	0.5094	0.6512	0.5948
UKF_*p*1 _(*λ* = 1)	0.7576		0.9355		0.8614		0.4375	0.5935	0.5121	0.5778	0.6744	0.6239
UKF_*p*1 _(*λ* = 5)	0.7500		0.9355		0.8439		0.3548	0.5455	0.4672	0.5814	0.7105	0.6456
UKF_*p*1 _(*λ* = 10)	0.7273		0.9063		0.8217		0.3226	0.4848	0.4156	0.6190	0.7368	0.6695

To consider the strength of the links, rather than setting it to 1, *e*_*ij*_ (in the indicator matrix ***E***) is set to different values. Large *e*_*ij*_ is used if the strength of the link from gene *j* to gene *i* is strong. For convenience, the UKF considering the strength of links is denoted as ${\text{UKF}}_{\hat{p}1}$. To compare the performance of ${\text{UKF}}_{\hat{p}1}$ with UKF_*p*1_, for ${\text{UKF}}_{\hat{p}1}$, the values of the second row in Equation (79) is multiplied by 5. The performance of ${\text{UKF}}_{\hat{p}1}$ using different *λ* is given in Table 5. It can be seen from Tables 4 and 5 that the performance of ${\text{UKF}}_{\hat{p}1}$ and UKF_*p*1_ is close when *λ* is small, e.g., *λ* = 0.1. In addition, the average TPR and FPR of ${\text{UKF}}_{\hat{p}1}$ is smaller than that of UKF_*p*1_ for all tested *λ* except for *λ* = 0.1. Hence, PPR is used to evaluate the performance of ${\text{UKF}}_{\hat{p}1}$ and UKF_*p*1_. Although the average PPR of ${\text{UKF}}_{\hat{p}1}$ and UKF_*p*1_ is close when the *λ* is large, e.g., *λ* = 10, the average PPR of ${\text{UKF}}_{\hat{p}1}$ is consistently higher than that of UKF_*p*1_. The results indicate that the inference accuracy of ${\text{UKF}}_{\hat{p}1}$ and UKF_*p*1_ are close when *λ* is very small or very large. The inference accuracy of ${\text{UKF}}_{\hat{p}1}$ outperforms UKF_*p*1_ when the appropriate strength of the link and parameter *λ* are used.

**Table 5:** **Effect of strength of the links using different *λ***

	**True positive rate**						**False positive rate**			**Positive predictive rate**		
**Filters**	**Min**		**Max**		**Avg**		**Min**	**Max**	**Avg**	**Min**	**Max**	**Avg**
${\text{UKF}}_{\tilde{p}1}$ (*λ* = 0.1)	0.7576		0.9677		0.8484		0.4688	0.7647	0.5713	0.5094	0.6512	0.5929
${\text{UKF}}_{\tilde{p}1}$ (*λ* = 0.5)	0.7576		0.9333		0.8468		0.4516	0.7059	0.5422	0.5385	0.6512	0.6057
${\text{UKF}}_{\tilde{p}1}$ (*λ* = 1)	0.7500		0.9032		0.8221		0.3750	0.5758	0.4953	0.5814	0.6842	0.6257
${\text{UKF}}_{\tilde{p}1}$ (*λ* = 5)	0.7273		0.8750		0.8220		0.3548	0.5000	0.4169	0.6098	0.7179	0.6684
${\text{UKF}}_{\tilde{p}1}$ (*λ* = 10)	0.7500		0.8750		0.8214		0.3226	0.5000	0.4143	0.6098	0.7368	0.6696

To consider the effect of false prior knowledge, the second row of the indicator matrix in Equation (79) is changed to [ 0,1,1,1,0,1,0,1], which conflicts with the truth. For convenience, we use ${\text{UKF}}_{\bar{p}1}$ to denote the UKF incorporating this false prior knowledge. In Table 6, the performance of ${\text{UKF}}_{\bar{p}1}$ with different *λ* is shown. It can be seen from Tables 4 and 6 that the average TPR of ${\text{UKF}}_{\bar{p}1}$ is smaller than that of UKF_*p*1_ when *λ* is small, e.g., *λ* = 0.1,0.5. In addition, the average FPR of ${\text{UKF}}_{\bar{p}1}$ is larger than that of UKF_*p*1_ when *λ* is large, e.g., *λ* = 5,10. Moreover, although the average PPR of ${\text{UKF}}_{\bar{p}1}$ is close to that of UKF_*p*1_ when *λ* is small, the average PPR of ${\text{UKF}}_{\bar{p}1}$ is consistently lower than that of UKF_*p*1_. Hence, as expected, the results indicate that the false prior knowledge will lead to worse inference result.

**Table 6:** **Effect of false prior information using different *λ***

	**True positive rate**						**False positive rate**			**Positive predictive rate**		
**Filters**	**Min**		**Max**		**Avg**		**Min**	**Max**	**Avg**	**Min**	**Max**	**Avg**
${\text{UKF}}_{\bar{p}1}$ (*λ* = 0.1)	0.7576		0.9667		0.8491		0.5000	0.7647	0.5933	0.5094	0.6279	0.5835
${\text{UKF}}_{\bar{p}1}$ (*λ* = 0.5)	0.7576		0.9355		0.8535		0.4839	0.7647	0.5962	0.5094	0.6279	0.5836
${\text{UKF}}_{\bar{p}1}$ (*λ* = 1)	0.7576		0.9333		0.8572		0.4839	0.7059	0.6001	0.5200	0.6279	0.5830
${\text{UKF}}_{\bar{p}1}$ (*λ* = 5)	0.6970		0.8125		0.7546		0.4194	0.5938	0.5000	0.5682	0.6486	0.6062
${\text{UKF}}_{\bar{p}1}$ (*λ* = 10)	0.5758		0.7576		0.6810		0.3226	0.5000	0.4066	0.5676	0.7059	0.6369

##### 

**Incorporating LASSO** The problem setup is the same as before except that the LASSO rather than the existing network information is used. The UKF incorporating LASSO is denoted as UKF_*p*2_.

As shown in Table 3, the average TP *#* and FP *#* of UKF_*p*2_ are lower than those of UKF and the average TN *#* and FN *#* of UKF_*p*2_ are higher than those of UKF. Hence, UKF_*p*2_ produces less links, including correct and incorrect ones. In addition, UKF_*p*2_ produces more nonlinks and missed links. It is consistent with the fact that the LASSO tends to provide a sparse solution. It can be seen from Table 3 that the average FPR of UKF_*p*2_ is lower than that of UKF and the average precision of UKF_*p*2_ is higher than that of UKF. Hence, by incorporating LASSO, the inference accuracy is improved.

**Figure 1:**
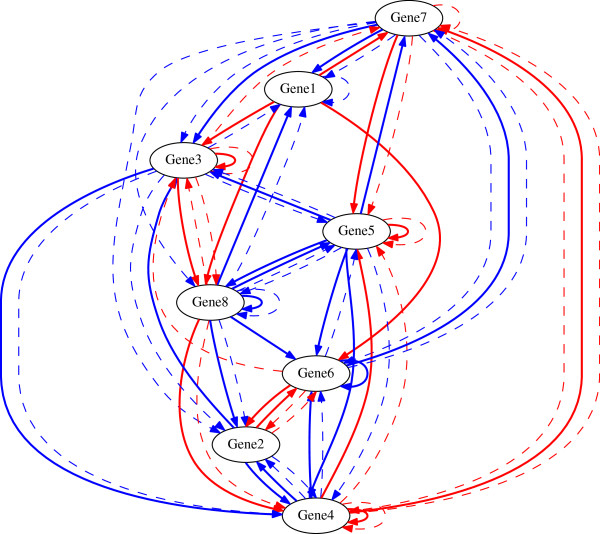
**Inferred regulations of UKF **_***p*** **2 **_**and true regulations.**

**Figure 2:**
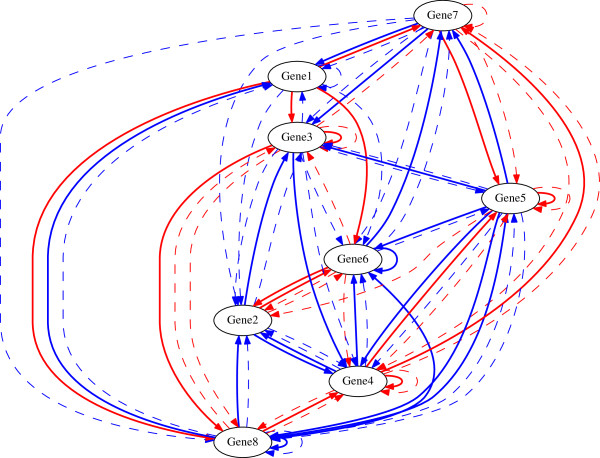
Inferred regulations of UKF and true regulations.

A representative inference result of UKF_*p*2_ and the true regulations are shown in Figure 1. For comparison, the inference result of UKF and the true regulations are shown in Figure 2. By comparing Figure 2 and Figure 1, it can be seen that UKF falsely predicts the nonlinks from gene1 to gene2, from gene3 to gene6, from gene4 to gene8, from gene5 to gene2, and from gene6 to gene4 while UKF_*p*2_ does not.

The performance UKF_*p*2_ with different *λ* is shown in Table 7. It is seen that the performance of UKF_*p*2_ and UKF is close when *λ* is small since only small regulation is imposed on the solution. When *λ* is large, the difference between UKF_*p*2_ and UKF is large. The average TPR and FPR of UKF_*p*2_ decrease with the increasing of the *λ*. The average PPR does not increase monotonicallly with the increasing of *λ*. Generally speaking, for different *λ*, UKF_*p*2_ is more sensitive than that of UKF_*p*1_. Although the performance of UKF_*p*2_ depends on *λ*, the average PPR of UKF_*p*2_ is consistently higher than that of UKF. Hence, roughly speaking, UKF_*p*2_ has better performance than UKF.

**Table 7:** **Comparison of UKF **_***p2 ***_** using different *λ***

	**True positive rate**						**False positive rate**			**Positive predictive rate**		
**Filters**	**Min**		**Max**		**Avg**		**Min**	**Max**	**Avg**	**Min**	**Max**	**Avg**
UKF_*p*2_ (*λ* = 0.1)	0.7576		0.9355		0.8304		0.4839	0.6970	0.5699	0.5306	0.6512	0.5914
UKF_*p*2_ (*λ* = 0.5)	0.6970		0.8710		0.7750		0.4194	0.5758	0.4902	0.5682	0.6585	0.6198
UKF_*p*2_ (*λ* = 1)	0.6970		0.7879		0.7335		0.4194	0.5000	0.4462	0.5897	0.6667	0.6355
UKF_*p*2_ (*λ* = 5)	0.4545		0.6667		0.5501		0.3226	0.4516	0.3791	0.5714	0.6471	0.6064
UKF_*p*2_ (*λ* = 10)	0.4545		0.5455		0.4800		0.2903	0.3871	0.3523	0.5556	0.6538	0.5920

##### 

**Incorporating the range constraint** The existing network information can be used to provide the rough range constraint of $\bar{x}$. A tight constraint is forced on the regulation coefficient *a*_*ij*_ when there is a small regulation possibility from gene*j* to gene*i* and a loose constraint is forced on the regulation coefficient with no prior information. In the simulation, for the coefficients corresponding to the zero elements in (79), the lower bound and the upper bound are set as -10 and 10, respectively. For the coefficients corresponding to the nonzero elements in (79), the lower bound and the upper bound are set as -0.1 and 0.1, respectively. The UKF incorporating the range constraint is denoted as UKF_*p*3_. As shown in Table 3, the average FPR of UKF_*p*3_ is lower than that of UKF and the average precision of UKF_*p*3_ is higher than that of UKF.

### 5.2 Yeast protein synthesis network

**Figure 3:**
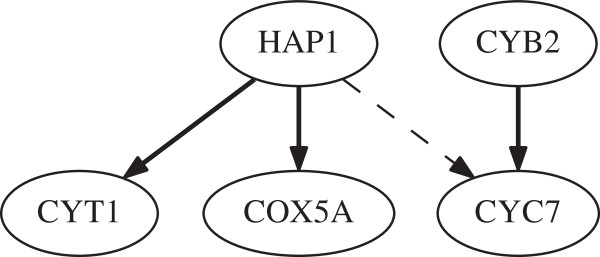
Pathway model of the five genes in yeast protein synthesis network.

In this section, time-series gene expression data of the yeast protein synthesis network is used. Five genes (HAP1, CYB2, CYC7,CYT1, and COX5A) of the yeast protein synthesis network are considered and 17 data points which can be found in [35] are collected. The regulation relationship between them has been revealed by the biological experiment and shown in Figure 3. The dashed arrow in Figure 3 denotes ‘repression’ and the solid arrow denotes ‘activation.’

**Figure 4:**
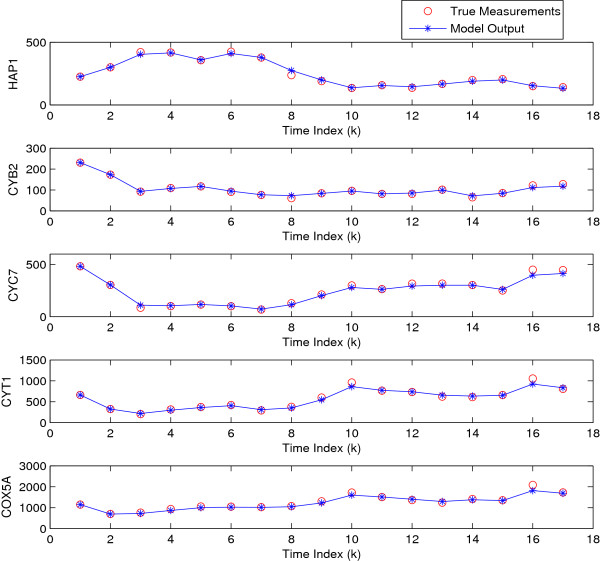
True gene expression and model output.

**Figure 5:**
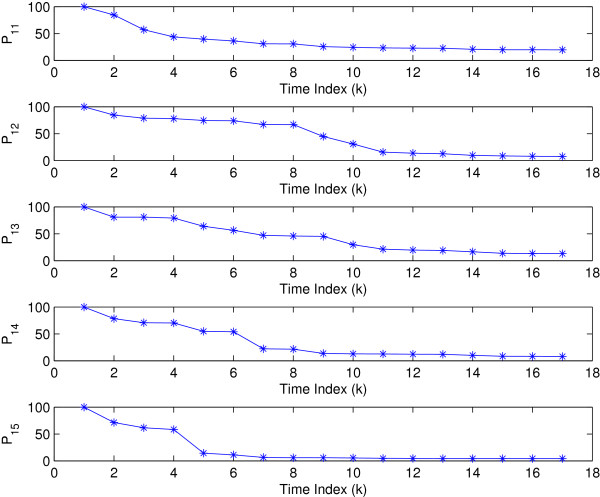
Variance of regulatory coefficients.

The GRN is inferred by the UKF and UKF_*p*2_. The predicted gene expressions using parameters estimated by UKF_*p*2_ and the true measured gene expressions are shown in Figure 4. It can be seen that the model output fits the measured data well. The variances of the regulatory coefficients of HAP1 (*P*_1*i*_ (1 ≤ *i* ≤ 5)) are shown in Figure 5. It can be seen that the filter converges since the variance *P*_1*i*_ approaches zero. The results for other regulatory coefficients are similar and not shown here. The evaluation of the inferred GRN by UKF and UKF_*p*2_ is shown in Table 8.

**Table 8:** **Inferred results of the UKF and UKF **_***p2***_

**Filters**	**True positives #**	**False positives #**	**True negatives #**	**False negatives #**
UKF	1	7	14	3
UKF_*p*2_	2	3	18	2
**Filters**	**TPR**		**FPR**	**Precision**
UKF	0.25		0.3333	0.1250
UKF_*p*2_	0.5000		0.1429	0.4000

By incorporating the sparsity constraint, UKF_*p*2_ provides much better inference results than UKF. As shown in Table 8, the TP *#* and TN *#* of UKF_*p*2_ are higher than those of UKF and the FP *#* and FN *#* are lower than those of UKF. In addition, it can be seen from Table 8, the FPR of UKF_*p*2_ is lower than that of UKF and the TPR and the precision of UKF_*p*2_ is higher than that of UKF.

## 6 Conclusions

In this paper, we have proposed a framework of employing the point-based Gaussian approximation filters which incorporates the prior knowledge to infer the gene regulatory network (GRN) based on the gene expression data. The performance of the proposed framework is tested by a synthetic network and the yeast protein synthesis network. Numerical results show that the inference accuracy of the GRN by the proposed point-based Gaussian approximation filter incorporating the prior information is higher than using the traditional filters without incorporating prior knowledge. The proposed method works for small- and medium-size GRNs due to the computational complexity considerations. It remains a future research topic how to adapt the proposed inference framework to handle large GRNs at reasonable computational complexity.

## Competing interests

All authors declare that they have no competing interests.
